# Supplementary material for: Group and individual level variations between symmetric and asymmetric DLPFC montages for tDCS over large scale brain network nodes
Source: Sci Rep. 2021 Jan 14;11:1271. doi: 10.1038/s41598-020-80279-0 (PMC7809198; doi:10.1038/s41598-020-80279-0)
Supplement: Supplementary file 1 — Supplementary Information. [file 41598_2020_80279_MOESM1_ESM.docx]

**Group and Individual Level Variations between symmetric and asymmetric DLPFC Montages for tDCS over Large Scale Brain Network Nodes**

Ghazaleh Soleimani^2^, Mehrdad Saviz^2*^, Marom Bikson^3^, Farzad Towhidkhah^2^, Rayus Kuplicki^1^, Martin P. Paulus^1^, Hamed Ekhtiari^1^

1. Laureate Institute for Brain Research (LIBR), Tulsa, OK, USA

2. Department of Biomedical Engineering, Amirkabir University of Technology, Tehran, Iran

3. Department of Biomedical Engineering, City College of New York of CUNY, NY, USA.

*Correspondence to [msaviz@aut.ac.ir](mailto:msaviz@aut.ac.ir)

**Supplementary Information**

**Table S1:** Group-averaged electric field intensity in [V/m] at large-scale brain networks

| Network | Hemisphere | Average (mean ± SD) EF [V/m] | | P value  (Corrected) |
| --- | --- | --- | --- | --- |
|  |  | F4-Fp1 | F4-F3 |  |
| Vis | Left | 0.0523 ± 0.01 | 0.0473 ± 0.01 | **0.0002*** |
|  | Right | 0.0532 ± 0.01 | 0.0488 ± 0.01 | **0.0026*** |
| SomMot | Left | 0.0972 ± 0.02 | 0.1016 ± 0.02 | 0.1765 |
|  | Right | 0.1014 ± 0.02 | 0.1017 ± 0.02 | 0.9284 |
| DorsAttn | Left | 0.0802 ± 0.02 | 0.0873 ± 0.02 | **0.0021*** |
|  | Right | 0.0896 ± 0.02 | 0.0885 ± 0.02 | 0.7708 |
| VentAttn | Left | 0.1257 ± 0. 03 | 0.1217 ± 0.03 | 0.3031 |
|  | Right | 0.1287 ± 0.03 | 0.1228 ± 0.03 | 0.1883 |
| Limbic | Left | 0.1955 ± 0.04 | 0.1540 ± 0.03 | **0.0000*** |
|  | Right | 0.1552 ± 0.03 | 0.1544 ± 0.03 | 0.8877 |
| ECN | Left | 0.1448 ± 0.03 | 0.1354 ± 0.03 | 0.0550 |
|  | Right | 0.1529 ± 0.04 | 0.1476 ± 0.04 | 0.3353 |
| DMN | Left | 0.1500 ± 0.03 | 0.1411 ± 0.03 | 0.0550 |
|  | Right | 0.1448 ± 0.03 | 0.1431 ± 0.03 | 0.2786 |

*Significant differences based on unpaired t-test after FDR correction

**Table S2:** Group-averaged electric field intensity in [V/m] in main nodes of large-scale brain networks

| Network | Node | Hemisphere | Average (mean ± SD) EF [V/m] | | P value  (Corrected) |
| --- | --- | --- | --- | --- | --- |
|  |  |  | F4-Fp1 | F4-F3 |  |
| VentAttn | ParOper | Left | 0.0817 ± 0.02 | 0.0785 ± 0.02 | 0.1878 |
|  | TempOccPar | Right | 0.0872 ± 0.02 | 0.0807 ± 0.01 | **0.0157*** |
|  | FrOperIns | Left | 0.1514 ± 0.03 | 0.1385 ± 0.03 | **0.0034*** |
|  |  | Right | 0.1583 ± 0.03 | 0.1490 ± 0.03 | **0.0479*** |
|  | PFC | Left | 0.2336 ± 0.05 | 0.2154 ± 0.05 | **0.0242*** |
|  | Med | Left | 0.1061 ± 0.1 | 0.1109 ± 0.1 | 0.1593 |
|  |  | Right | 0.0995 ± 0.02 | 0.0986 ± 0.1 | 0.7921 |
| Limbic | OFC | Left | 0.2474 ± 0.05 | 0.1935 ± 0.04 | **0.0000*** |
|  |  | Right | 0.2114 ± 0.04 | 0.2055 ± 0.04 | 0.3533 |
|  | TempPole | Left | 0.1342 ± 0.03 | 0.1073 ± 0.02 | **0.0000*** |
|  |  | Right | 0.1104 ± 0.02 | 0.1158 ± 0.02 | 0.0631 |
| ECN | TempPar | Left | 0.0635 ± 0.01 | 0.0704 ± 0.02 | **0.0005*** |
|  |  | Right | 0.0732 ± 0.02 | 0.0712 ± 0.01 | 0.4020 |
|  | PFC | Left | 0.1937 ± 0.04 | 0.1661 ± 0.04 | **0.0000*** |
|  |  | Right | 0.1728 ± 0.04 | 0.1650 ± 0.04 | 0.4020 |
|  | PcunCing | Left | 0.0688 ± 0.01 | 0.0544 ± 0.01 | **0.0000*** |
|  |  | Right | 0.0650 ± 0.01 | 0.0537 ± 0.01 | **0.0000*** |
| DMN | TempPar | Left | 0.0902 ± 0.02 | 0.0783 ± 0.01 | **0.0000*** |
|  |  | Right | 0.0935 ±0.02 | 0.0884 ± 0.02 | 0.0557 |
|  | PFC | Left | 0.2019 ± 0.05 | 0.1966 ± 0.05 | 0.4249 |
|  |  | Right | 0.2000 ± 0.05 | 0.1985 ± 0.05 | 0.7978 |
|  | PcunPCC | Left | 0.0595 ± 0.01 | 0.0500 ± 0.01 | **0.0000*** |
|  |  | Right | 0.0629 ± 0.01 | 0.0490 ± 0.01 | **0.0000*** |

*Significant differences based on unpaired t-test after FDR correction


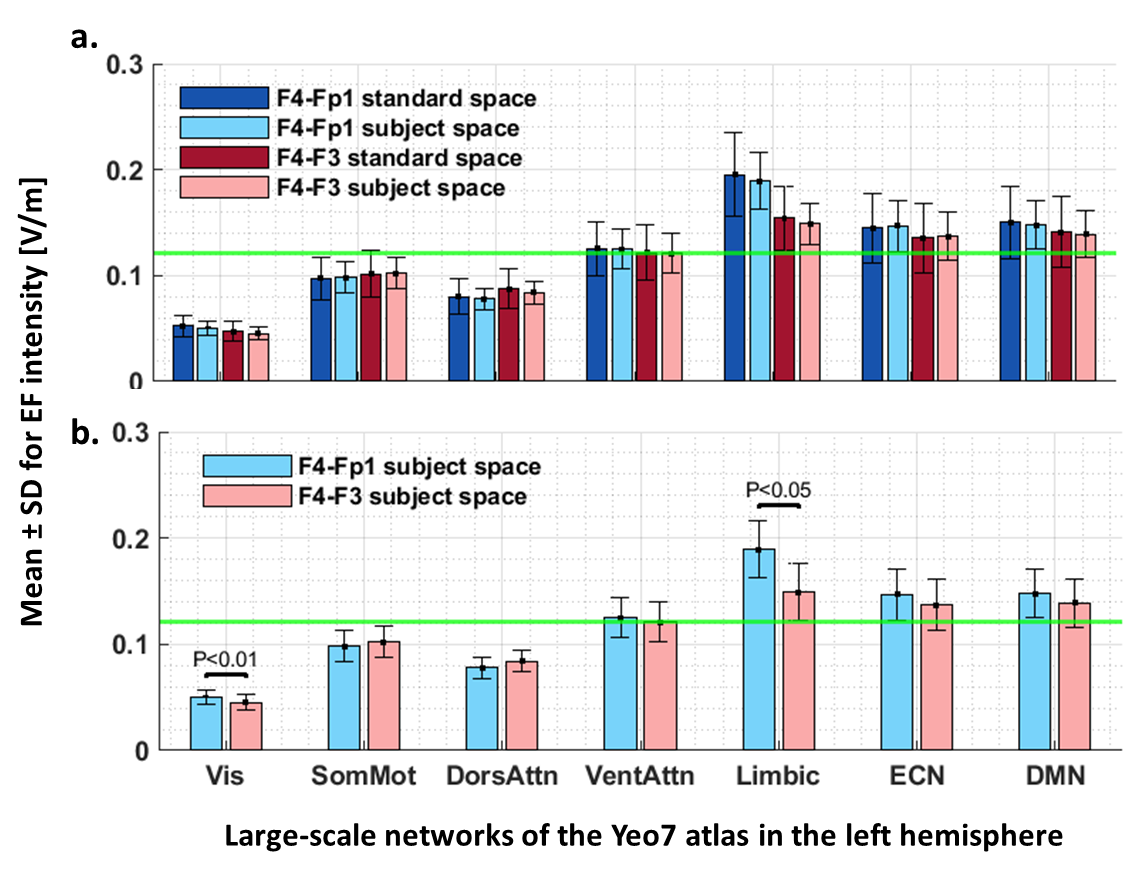


**Figure S1:** Parcellation of the average EF intensity at a current of 2mA generate by each montage in both standard-space and subject-space. Bars show mean value and error bars show SD of the EF intensity in volt per meter across 66 participants in each large-scale network of Yeo7-2011 atlas only in the left hemisphere. **a.** both montages and both spaces. **b.** between montage differences for the results in subject-space. Significant differences between the two montages are shown above the bars based on the t-test with FDR correction threshold at P < 0.05. The horizontal green line indicates EF threshold (50% minimum value of peak EFs in whole-brain analysis across the subjects). **Blue**: F4-Fp1 montage, **Red**: F4-F3 montage, **Light colors**: subject-space, **Dark colors**: standard-space.


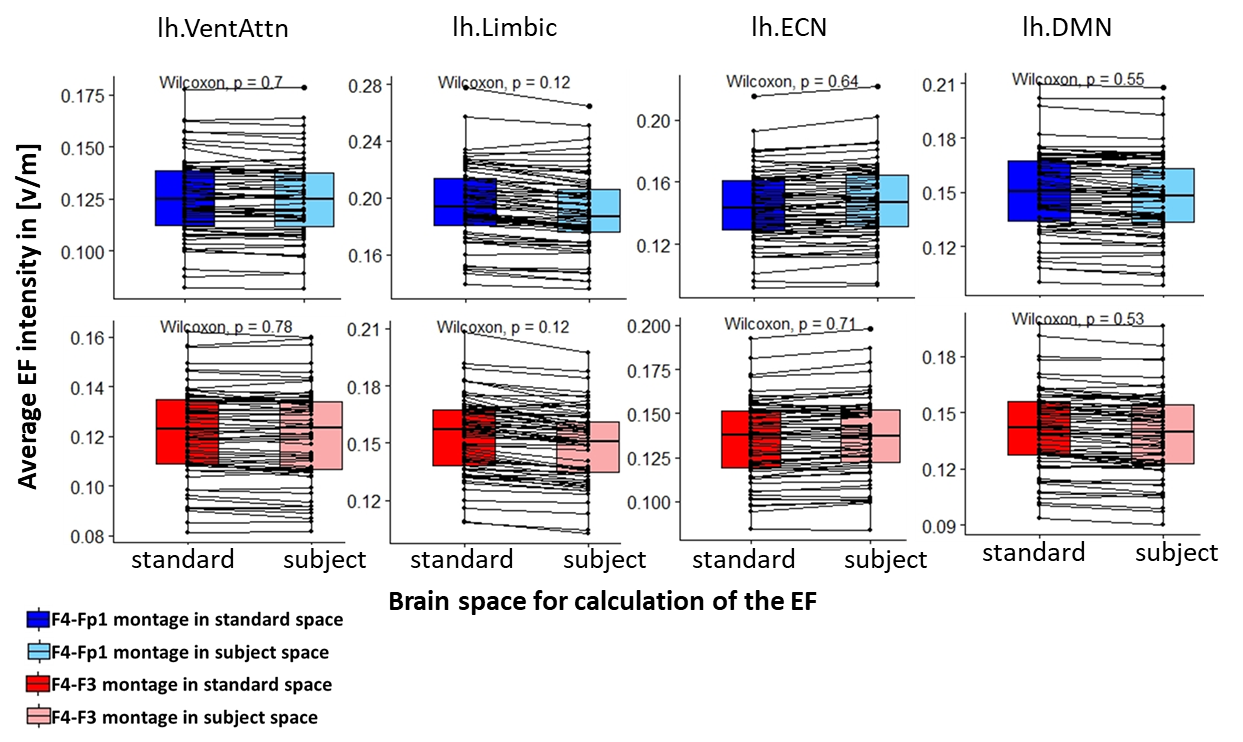


**Figure S2:** Averaged EF intensity calculated for all 66 participants inside the large-scale brain networks in the left hemisphere; first row for F4-Fp1 montage (dark blue: in standard-space, light blue: in subject-space) and second row for F4-F3 montage (dark red: in standard-space, light red: in subject-space). Results are visualized for the networks with averaged EFs above the threshold. Box plot showing the effects of brain space (subject-space or transformation to standard-space) on averaged EF intensity. Dots represent the data for each subject. Statistical results for between space differences are reported above boxplots.
